# Caste development and sex ratio of the Ryukyu drywood termite *Neotermes sugioi* and its potential mechanisms

**DOI:** 10.1038/s41598-021-94505-w

**Published:** 2021-07-22

**Authors:** Y. Miyaguni, A. Agarie, K. Sugio, K. Tsuji, K. Kobayashi

**Affiliations:** 1 Global Education Institute, University of the Ryukyus, Okinawa, 903-0213 Japan; 2 Department of Environmental Science and Conservation Biology, United Graduate School of Agricultural Sciences, Kagoshima University, Kagoshima, 890-8580 Japan; 3 Graduate School of Education, University of the Ryukyus, Nishihara, Okinawa 903-0213 Japan; 4 Entomological Laboratory, Faculty of Agriculture, University of the Ryukyus, Okinawa, 903-0213 Japan; 5 Field Science Education and Research Center, Hokkaido Forest Research Station, Kyoto University, 553 Tawa, Shibecha-cho, Kawakami-gun, Hokkaido, 088-2339 Japan

**Keywords:** Evolution, Evolutionary developmental biology, Behavioural ecology, Evolutionary ecology

## Abstract

Sex allocation is one of the most studied traits in evolutionary biology because its theoretical predictions match the empirical data. Here, using the Ryukyu dry-wood termite *Neotermes sugioi*, we investigated several factors that could bias the sex allocation in three populations (Okinawa, Ishigaki/Iriomote, and Yonaguni). Our survey showed that there were more queen-only colonies than king-only colonies in these populations, suggesting a longer lifespan of the queens than that of the kings. In this condition, sex-asymmetric reproductive value (SRV) theory predicts female bias, because even after the short-lived kings die, the long-lived queens can continue reproduction with their sons. However, sex allocation in this species seemed to be biased toward males. Furthermore, we examined the possibility of intrasexual competition among siblings (ICS). If ICS is the cause of the bias, the allocation is expected to change depending on the total investment in sexual offspring. However, the biomass of both male and female alates increased linearly with the increase in the total biomass of the alates in these populations. Thus, neither the SRV nor the ICS theory could explain the male-biased sex ratio of *N. sugioi*. On the basis of these results, we discuss the remaining possibilities in this species.

## Introduction

Sex allocation is one of the best-studied cases of adaptation, as theoretically predicted optima match well with the observed values. Most organisms show equal allocation to male and female offspring^1^; however, significant deviations from equal allocation have been reported in various taxa^2^. In termites, these deviations could be explained by intrasexual competition within siblings (ICS) or sex-asymmetric reproductive value (SRV)^3,4^ that is called as sex-asymmetric inbreeding in the previous studies^5^. The ICS is known as local mate competition^6^, local resource competition^7^, and local resource enhancement^8^, where only individuals of either sex compete or cooperate with their relatives. When ICS occurs, it is expected that adaptive allocation changes depending on the total investment in sexual offspring^2,9,10^. For the colonies with large amounts of resources, the overproduction of non-competing or cooperating sex is adaptive because their offspring must compete or cooperate with relatives more frequently compared to the offspring produced by the colonies with small amount of resources. As the result, such productive colonies produce a constant number of competitive or non-cooperative sex irrespective of the available amount of resources. Thus, the theory of ICS predicts a constant production of relatively infrequent sex, independent of the total alate production of the colonies. The theory of SRV predicts that the sex that mates with their offspring within the colony after the original mating partner dies will be produced more than the other sex, because such longer-lived sex can transfer its genes to future generations more than the shorter-lived sex can^5,11^. Since the sex found more frequently when examining termite colonies in the field are thought to be relatively more long-lived than the other, we can expect that such sex will be produced more than the other sex based on the SRV theory. Thus, examination of the sexual differences in competitiveness and longevity can reveal which factors are more important for the sex allocation of each termite species with unequal sex ratios.

A puzzling relationship between colony composition and sex allocation has been reported in some *Neotermes* species (Kalotermitidae). In this genus, a colony is founded by a monogamous pair of alates which become the king and the queen^12^. After the death of one of the founders, one of their offspring of the same sex as the dead founder develops into a neotenic reproductive (secondary king or queen) and inherits the reproductive role in the colony^13–16^. Interestingly, in *N. connexus*, *N. papua*, and *N. sugioi*, all neotenic reproductives are males^14–16^, which implies that the females have no chance of being secondary queens but to disperse as alates^12^. Thus, the primary queen will mate with the secondary neotenic king (her son), whereas on the other hand, due to the presumable obligate pre-copulatory dispersal in female alates, the primary king does not have an opportunity to mate with the secondary queen (his daughter) and dies without reproduction after the death of his partner. In this condition, the SRV theory predicts female-biased sex allocation^5^. However, in the colonies of *N. connexus* and *N. sugioi*, more male alates than female alates were found^14,17^. It remains to be seen if this inconsistency can be explained by the current sex allocation theories.

There are several possible solutions to this puzzle in *Neotermes* termites. The first is that sexual differences in body size have the potential to translate the observed numerical ratio into the predicted sex allocation. In other words, if females are heavier than males, the male-biased numerical ratios that have been reported in the previous studies may become female-biased investment ratios. Although there is no evidence of sexual differences in body size of *N. sugioi* in a previous study^18^, since the number of samples is small, it is necessary to reinforce the data significantly. Second, the strong selective pressure of ICS can bias sex allocation toward males despite SRV. In this case, it is expected that the biomass of male alates remains constant even if the total alate biomass produced by the colony is large while production of female alates increases linearly with the total alate biomass^2,9^. As another indicator of the amount of available resources, colony size could be used. Thus, it is expected that larger colonies produce alates at a more male-biased ratio. The third possible solution is that female alates could become the replacement queens in their natal colonies, and they are then called ‘adultoids’, which completely overturns the premise of the SRV theory. In *Neotermes* termites, secondary reproductives are classified into neotenics derived from immature individuals and adultoids derived from dealate imagoes^15,19^. As the founding individuals (primary king and queen) are also derived from dealate imagoes, they are distinguishable from neotenics but not from adultoids. Thus, some of the mature reproductives reported as founders in previous studies might be adultoids that are in fact offspring of the founders. In cases when the female adultoid is functional, the longevity of females is shorter than that of males, and the queen is more frequently replaced by the female adultoid than the king replaced by the male adultoid, the SRV theory predicts male-biased allocations. In this situation, assuming no sex difference in time to develop into queen or king, we can expect that field colonies lack the queen more frequently than the king, because of the frequent turnover of the queen position.

In the present study, we studied the Ryukyu dry-wood termite *N. sugioi* (new *Neotermes* species recently separated from *N. koshunensis*)^18^ in which colonies are monogamous and almost all neotenics are male^12,16,20^. We surveyed the numerical sex ratios, body sizes of alates, and colony sizes in colonies sampled from three populations (Okinawa, Ishigaki/Iriomote, Yonaguni) to clarify whether sex allocation of alates is biased toward males in this species. We also examined whether the ICS and SRV theories can explain the biased allocation. Furthermore, we surveyed the numerical sex ratios of pseudergates, soldiers, and nymphs to clarify at which point during their development stages does the bias occur. Based on these results, we discussed the remaining possibilities to explain this inconsistency.

## Results

### Numerical sex ratio

**Figure 1:**
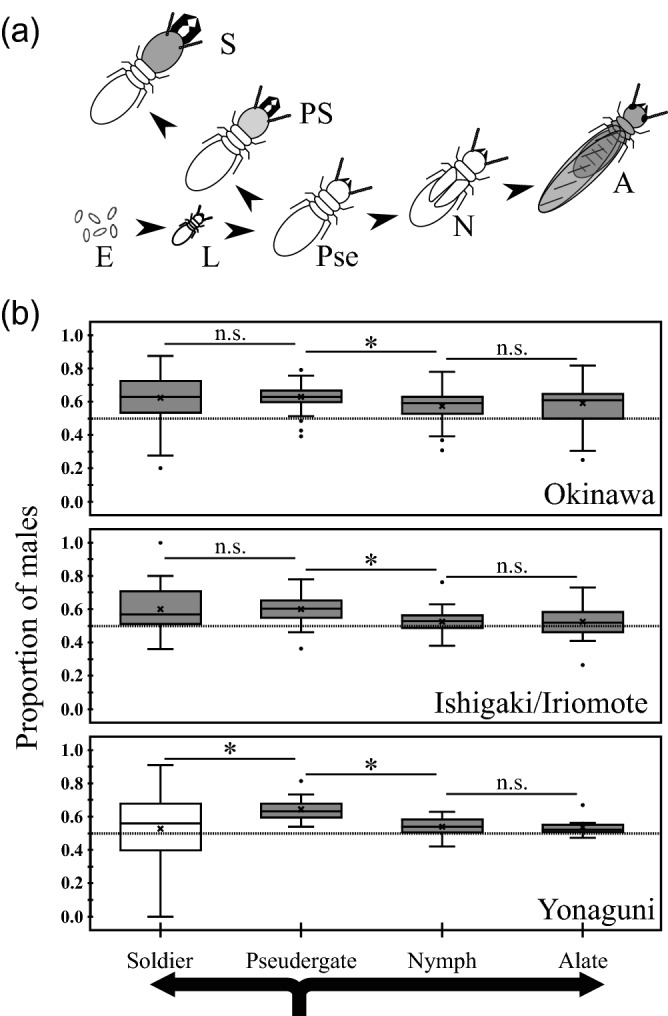
Relationship between caste developmental pathway and numerical sex ratios in the three populations. (**a**) The caste developmental pathway of *Neotermes sugioi*^26^. *E* egg, *L* larva, *Pse* pseudergates, *PS* pre-soldier, *S* soldier, *N* nymph, *A* alate. (**b**) Comparisons of numerical sex ratios between castes. In each box, the cross symbol indicates the mean, bar in the box indicates the median, and the box top and bottom indicate the first and third quartiles, respectively. The asterisk next to each bar between boxes indicates *p* < 0.05 (glmm analysis with sequential Bonferroni), and "n.s." indicates “not significant”, i.e., *p *≥ 0.05. The gray boxes indicate deviations from equal sex ratios at *p* < 0.05 (Wald tests in glm analysis with sequential Bonferroni), and the white boxes indicate p ≥ 0.05.

In each population, the total number of male individuals was higher than that of female individuals, regardless of caste (binomial tests for Okinawa alates, total female:male individuals = 2,427:3,468, *p* < 0.0001; for Okinawa nymphs, 9,391:14,305, *p* < 0.0001; for Okinawa pseudergates, 12,362:21,687, *p* < 0.0001; for Okinawa soldiers, 1,126:1,984, *p* < 0.0001, for Ishigaki/Iriomote alates, 2,951:3,144, *p* = 0.0139; for Ishigaki/Iriomote nymphs, 8,143:8,779, *p* < 0.0001; for Ishigaki/Iriomote pseudergates, 8,365:13,226, *p* < 0.0001; for Ishigaki/Iriomote soldiers, 1,126:1,984, *p* < 0.0001, and for Yonaguni alates, 2,951:3,144, *p* = 0.0139; for Yonaguni nymphs, 8,143:8,779, *p* < 0.0001; for Yonaguni pseudergates, 8,365:13,226, *p* < 0.0001; for Yonaguni soldiers, 1,126:1,984, *p* < 0.0001; Supplementary Table 1). The proportion of males in each colony was clearly biased towards males in all of the castes (Fig. 1b; Wald tests in the glm analysis with sequential Bonferroni all deviated from the equal ratio, *p* < 0.0001), except for marginally male-biased alates in Ishigaki/Iriomote (*z* = 2.4717, *p* = 0.0403) and Yonaguni (*z* = 2.4018, *p* = 0.0326), and no bias was detected in Yonaguni soldiers (*z* = − 0.3303, *p* = 0.7412). Compared to the nymphs, the pseudergates showed more male-biased sex ratios (Fig. 1b; glmm analysis for the data of each population with sequential Bonferroni, Okinawa, *z* = 10.6800, *p* < 0.0001; Ishigaki/Iriomote, *z* = 17.6840, *p* < 0.0001; Yonaguni, *z* = 4.4210, *p* < 0.0001). In the Okinawa and Ishigaki/Iriomote populations, the pseudergates showed a similar sex ratio to that of the soldiers, but in the Yonaguni population, the pseudergates were more male-biased than the soldiers (Fig. 1; glmm analysis with sequential Bonferroni, Okinawa, *z* = 0.9411, *p* = 0.6940; Ishigaki/Iriomote, *z* = − 2.2610, *p* = 0.1185; Yonaguni, *z* = − 2.8640, *p* = 0.0251). In all populations, the nymphs showed a similar sex ratio to that of the alates (Fig. 1; glmm analysis with sequential Bonferroni, Okinawa, *z* = 1.1560, *p* = 0.7440; Ishigaki/Iriomote, *z* = − 0.6086, *p* = 0.5430; Yonaguni, *z* = 1.5780, *p* = 0.4600). No significant effect of the colony size on the proportion of males was detected in any caste of any population (Supplementary Fig. 1). Note that the data included two colonies with poor food availability (C171 and C172), even in which males were more abundant than females in all castes (Supplementary Table 1).

### Measurement of alate body size and sex allocation

In the Okinawa and Ishigaki/Iriomote populations, the head width of male alates was 0.8% and 0.7% larger than that of female alates, respectively (Supplementary Table 2). No sexual differences in dry weight were found between these two populations (Supplementary Table 2). In the Yonaguni population, no sexual difference was detected in head width, but the dry weight of males was 3.3% lower than that of females (Supplementary Table 2).

**Figure 2:**
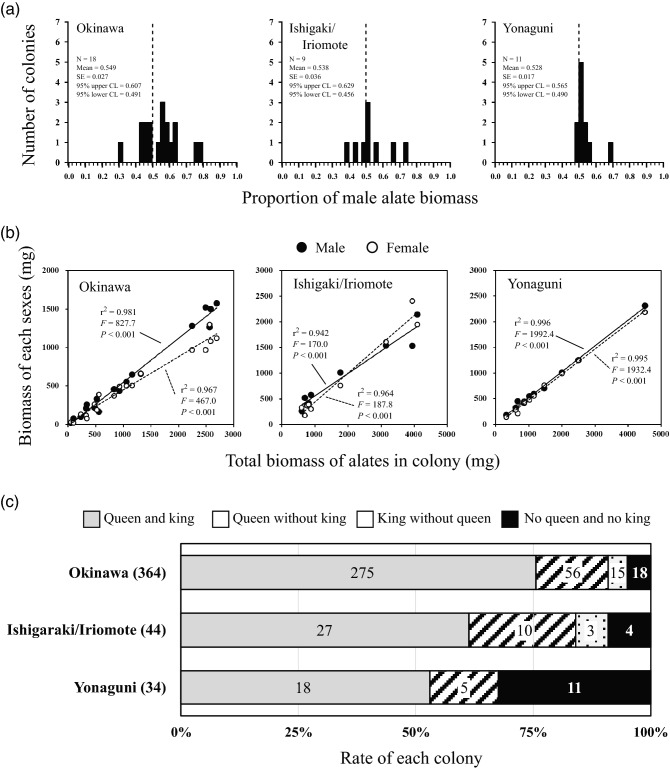
Characteristics of sex allocation and results of the test for intrasexual competition among siblings (ICS) and sex-asymmetric longevity corresponding to sex-asymmetric reproductive value (SRV) in the three populations. (**a**) Histogram of the proportion of biomass of male alates in the colonies of the three populations. (**b**) Plot analysis of the model of ICS. (**c**) Pattern-frequency of reproductives in the three populations. Solid lines (males) and dashed lines (females) in (**b**) show the correlations between the total biomass of alates in a colony and the biomass of alates of each sex in a colony, and numerals on the graph (**c**) indicate the number of colonies. *CI* confidence interval.

In the Okinawa population, alate biomass as an index of sex allocation was calculated by multiplying the mean dry weight by the number of alates for each sex, and it was biased toward males (proportion of male biomass = 0. 549 ± 0.027 (mean ± SE), paired *t*-test, *n* = 18, *t* = 2.445, *p* = 0.026; Fig. 2a). Such biased sex allocation was not detected in the Ishigaki/Iriomote (proportion of male biomass = 0. 538 ± 0.036 (mean ± SE), paired *t*-test, *n* = 9, *t* = 0.079, *p* = 0.939), and Yonaguni populations (proportion of male biomass = 0. 528 ± 0.018 (mean ± SE), paired *t*-test, *n* = 11, *t* = 1.835, *p* = 0.097). In all the populations, both male and female alate biomass increased linearly with the increase in total alate biomass in the colonies (Fig. 2b; Supplementary Table 3).

### Estimating the lifespans of kings and queens

Out of a total of 364 colonies sampled from the Okinawa population, 56 had a queen but no king, whereas 15 had a king but no queen. Out of a total of 44 colonies from the Ishigaki/Iriomote population, ten had a queen but no king, while three had a king but no queen. Out of a total of 34 colonies from the Yonaguni population, five had a queen but no king, while no colonies with a king but no queen were found (Fig. 2c). Thus, in all the populations, colonies with a queen but no king tended to be more frequent than those with a king but no queen (binomial tests, *p* < 0.0001 for Okinawa, *p* = 0.0923 for Ishigaki/Iriomote, *p* = 0.0625 for Yonaguni). There was no difference in the ratio of these two colony types among the populations (Fig. 2c; chi-squared test, χ^2^ = 1.369, d.f. = 2, *p* = 0.504).

## Discussion

In *Neotermes sugioi*, male bias of colony members was common in most castes of the studied populations (Fig. 1 and Supplementary Table 1). Our survey did not provide solid evidence that this bias could be explained by either the ICS or the SRV theory. If biased sex allocation is caused by ICS, it would be expected that allocation change depending on the available resources for the colony, which results in a strategy of constant investment into the rare sex^2,9,10^. However, colony size-dependent allocation predicted in the ICS theory was not detected in the present study; most colonies produced more male than female alates, regardless of colony productivity (Fig. 2b). These results clearly showed that the ICS mechanism is not an important factor for male-biased sex allocation in this species. Furthermore, the frequency of reproductives in the field colonies suggested that the queens live longer than the kings, and thus, the SRV theory, which predicts female bias, cannot explain the observed male-biased sex ratio in alates (Fig. 2c). If male-biased sex allocation in *N. sugioi* was caused by SRV, male reproductives have a longer lifespan than that of female reproductives. However, our data, along with previously published data^12,20^, revealed that the frequency of field colonies without queens but with kings was lower than that of colonies without kings but with queens in all studied populations (Fig. 2c). This result suggested that the lifespan of female reproductives is longer than that of males in all studied populations. Therefore, the SRV theory could not adequately explain the male-biased sex ratio in this species. Currently, the available information cannot solve the puzzle of selective advantages of the male-biased sex ratio in *N. sugioi*. In the case of SRV occurred in other termites, SRV is caused by the asexual queen succession (AQS) system where workers, soldiers and alates are produced sexually while numerous neotenic (secondary) queens arise through thelytokous parthenogenesis^11,21–24^. This system allows the primary queen in the colony to be genetically longer-lived than the primary king. As a result, the sex ratio is biased towards females. If the males of *N. sugioi* can reproduce their sons (next kings) asexually, as the queens asexually reproduce next queens in the AQS species, it may explain the puzzling male-biased sex ratio in this species. More detailed studies including genetic research are required to reveal the sexual differences in lifespan in *N. sugioi*.

The data from the present study did not match the predictions of the sex ratio theory. Interestingly, we found that the proportion of males was high in pseudergates and soldiers, but lower in nymphs, which is the differentiation stage for non-reproductive and reproductive individuals, respectively (Fig. 1). In other words, the sex ratio, which was biased during the developmental stage (pseudergate), seemed to be coming close to the equal ratio when differentiating into the reproductive line (nymph-pathway), but remained biased when differentiating into the non-reproductive line (soldier-pathway), except in the Yonaguni population (Fig. 1). These results indicated that the sex ratio, which was distorted by some factors in the early stages of development, approached 1:1 in mature individuals (alates), which are the focus of the sex allocation theory. If so, the male-biased sex ratio in *N. sugioi* may be a result of the conflict between distorting and returning factors during the developmental process, and not a result of the adaptation of individuals or colony to the environmental factors associated with their life history traits. In this case, the sex ratio of *N. sugioi* would be male-biased, even in eggs.

Kalotermitidae termites are often assumed to have an equal sex ratio compared to other termite families^13,19,25–27^. However, in some genus of Kalotermitidae, some species show male-biased sex ratios at pseudergates or young larval stage while other species of the same genus show equal sex ratios^14,15,19,28,29^, and such biases have been found in a wide range of the world: *Incistermes schwarzi* in southeast Florida^30^; *Cryptotermes dudleyi* in the northwest coast of Penang Island^29^; *N. connexus* in Honolulu^14^; *N. papua* in Bogia District, Papua New Guinea^15^. In the species *C. domesticus*, pseudergates shows equal sex ratios in Darwin Harbour, Australia, but male-biased sex ratios at Kundong, China^26,28^. Interestingly, similar to our results of *N. sugi*oi, the male-biased sex ratio in pseudergates was altered toward equal sex ratio or female-biased sex ratio through caste differentiation to reproductive lineages, such as nymphs and alates in *Cryptotermes* and *Incistermes*^29–31^. These knowledges suggest that the trait of the male-biased sex ratio during development but approaching to 1:1 at alates has evolved repeatedly in Kalotermitidae. The repeated evolution implies that a very simple factor is responsible for this evolution. To reveal the factor, further research is required.

The caste differentiation to alates in termites is sometimes affected by the environmental condition with the seasonal changes^17,32–35^ and the colony-level condition such as the food availability of colony^36–38^. For *N. sugioi*, the previous study showed that the proportion of males in alates in the Okinawa Island were maintained at approximately 0.6 for most of alate flight season^17^, which is consistent with our results of sex ratio in alates (Fig. 1; Supplementary Table 1). Thus, the alate sex ratio in this termite will be not or hardly affected by environmental conditions with the seasonal change. In this study, only two colonies (C171 and C172) may have a poor food availability, but even such colonies showed a male-biased sex ratio (Supplementary Table 1). However, due to the small number of samples in our data, the effect of colony conditions on the sex ratios needs to be tested in the future.

Some genetic elements subvert the laws of Mendelian segregation to promote their own transmission relative to the rest of an individual's genome^39,40^; these elements were called "selfish genetic elements (SGEs)" because they neglect the benefits of their host individuals. Therefore, the presence of SGEs results in a non-adaptive sex ratio of individuals in some cases^41,42^. If such sex ratio-distorting factors and their resistance factors existed in *N. sugioi*, and the strength of the conflict between these factors varied among the populations, we could verify this scenario through interpopulation mating experiments. Mating between strong and weak conflict populations is expected to produce eggs with more extreme sex ratios^43^. Thus, interpopulation mating experiments would be helpful for detecting sex-ratio distortions in *N. sugioi*.

Our research showed that *N. sugioi* is a challenging material for understanding the complex mechanisms that regulate the sex ratio and sex allocation of organisms. At least to the extent that we investigated, the sex allocation theories based on the SRV and ICS mechanisms could not explain the male-biased sex ratio in *N. sugioi*. Their male-biased sex ratio might be caused by other mechanisms that are neither ICS nor SRV. SGEs could be a possible explanation for this, but further investigation is needed to verify this possibility.

## Materials and methods

### Numerical sex ratio

In the period from 2010 to 2018, we collected a total of 102 colonies of *N. sugioi* from three populations: Okinawa population (62 colonies from Okinawa Island), Ishigaki/Iriomote population (29 colonies from Ishigaki Island and Iriomote Island), and Yonaguni population (11 colonies from Yonaguni Island) in the Ryukyu Archipelago, Japan (Supplementary Table 1). Each population is geographically separated by the ocean; the minimum distance is approximately 100 km (Ishigaki/Iriomote to Yonaguni) and a maximum of 520 km (Okinawa to Yonaguni population). The Okinawa population is the northern and eastern limit of the distribution of this termite^44^ and the Yonaguni population is estimated as the origin location of this species^18^. Sampling was mainly done from May to August during a season of dispersal flight in this termite^34^, but some colonies were collected during the off-season of flight, January and February (Supplementary Table 1). Previous studies^15^ and our preliminary experiments have revealed that in all populations, these termites show a male-specific neotenic system (Supplementary Table 4). In this species, most colonies live in a single dead branch of a living tree^12^. So, when we sample their colonies, to obtain the entire colony, the branches with termite colonies were cut into long pieces and confirmed that there was no nest-gallery of the termite on the cut surface at the root side of the branch. In this procedure, most colonies had abundant food availability until they were collected^12^. There were two colonies (C171 and C172 in Supplementary Table 1) where the nest area extended to the roots of the trees, and it may have poor food availability.

The collected nested branches were transported to the laboratory. Then, they were cut into small blocks, and all colony members (including reproductives) were collected and classified into the following castes^16,45^: adult queen and adult king, neotenic king (secondary king), pseudergates (older larvae, functional worker caste), nymphs (first nymphs and pre-alate nymphs), alates, and soldiers (pre-soldiers and soldiers). *N. sugioi* shows a linear caste developmental pathway: pseudergates can develop into alates through the nymph caste, and soldiers develop from pseudergates^19,45^. Sexual dimorphism in sternite structure was used for sexing under a stereomicroscope^46^. For alates and soldiers (pre-soldiers and soldiers), we sexed all individuals in all colonies. For pseudergates and nymphs, we sexed at least 190 individuals in mature colonies and all individuals in early or young colonies. The numerical sex ratio was expressed as the proportion of males in all alates. Colony size was recorded for 90 colonies.

We performed binomial tests to evaluate whether the numerical sex ratio is biased in each of the three populations. We also performed glm analysis with a binomial error terms and a logit link function to evaluate whether the numerical sex ratio is biased when each of colony is regarded as a repeat. To compare the numerical sex ratios between castes, glmm analysis was performed with a binomial error terms, a logit link function and a random colony identity effect in each population, using "glmmML" package (https://doi.org/10.1016/j.csda.2011.06.011) in R version 4.0.2.

### Measurement of termite body size and sex allocation

From the 102 already described colonies, 18 colonies of the Okinawa population, nine colonies of the Ishigaki/Iriomote population, and 11 colonies of the Yonaguni population were used to clarify the sexual difference in body size of alates and the sex allocation of colonies. For each colony of the Okinawa population, we measured the body size of at least three males and three females (maximum ten males and ten females). In the Ishigaki/Iriomote and Yonaguni populations, we measured at least ten males and ten females in each colony. These samples were kept separately in a freezer (− 40 °C) in 2 mL tubes until measurements. After the samples were thawed, their head width^47^ was measured using a digital camera attached to a stereomicroscope (Olympus E-System E-330; Olympus Corporation, Tokyo, Japan). Then, the samples were dried at 50 °C for 48 h and their dry weights were measured using an electronic balance (with a minimum value of 0.1 mg). Sexual differences in body size were tested using paired *t*-test, where the paired values were the means of males and females of each colony. The investment (biomass) of the colony in each alate sex was estimated by multiplying the mean dry weight and the number of alates for each sex. Sex allocation was expressed as the proportion of the investment to males in the total investment. Because the ICS theory predicts constant allocation toward the rarer sex, to evaluate whether the allocation to each sex changes depending on the total biomass of alates in each colony, linear regression analysis was performed in each of the three populations.

### Estimating the lifespans of reproductive kings and queens

Typically, *N. sugioi* colonies are headed by a monogamous pair, which includes a king and a queen^12,20^. If there is a difference in lifespan between the king and the queen, an imbalance is expected between the number of colonies with a king but without a queen and those with a queen but without a king. We assessed their frequencies in field colonies using our previously published sampling data^12^ and the results of a previous study reporting the reproductive composition of field colonies of this species^20^. Here, the king and the queen refer to dealate reproductives (adultoids), and not to neotenic reproductives. For the Okinawa population, field sampling data for 84 colonies and 280 colonies were available from two previous studies^12,20^. In the present study, we sampled 62 field colonies in the Okinawa population, for which the frequencies of kings and queens have been reported in a previous study^12^. To investigate the frequencies of kings and queens in the Ishigaki/Iriomote and Yonaguni populations, we collected 44 and 34 colonies from these populations, respectively, in the period from June 2014 to July 2020. The collected colonies were brought to the laboratory, and colony members, including reproductives, were collected using the methods described above. Some of these colonies were used in the sex ratio surveys and body size measurements described above (29 colonies of the Ishigaki/Iriomote population and 11 colonies of the Yonaguni population). The imbalance in the number of colonies with a king but without a queen to those with a queen but without a king was tested by Fisher's exact test in every population, and the difference in the ratio of these colonies among populations was tested using the χ^2^ test.

## Supplementary Information

Supplementary Information.
