# Publisher Correction: Caste development and sex ratio of the Ryukyu drywood termite *Neotermes sugioi* and its potential mechanisms

**DOI:** 10.1038/s41598-021-95744-7

**Published:** 2021-08-04

**Authors:** Y. Miyaguni, A. Agarie, K. Sugio, K. Tsuji, K. Kobayashi

**Affiliations:** 1 Global Education Institute, University of the Ryukyus, Okinawa, 903‑0213 Japan; 2 Department of Environmental Science and Conservation Biology, United Graduate School of Agricultural Sciences, Kagoshima University, Kagoshima, 890‑8580 Japan; 3 Graduate School of Education, University of the Ryukyus, Nishihara, Okinawa 903‑0213 Japan; 4 Entomological Laboratory, Faculty of Agriculture, University of the Ryukyus, Okinawa, 903‑0213 Japan; 5 Field Science Education and Research Center, Hokkaido Forest Research Station, Kyoto University, 553 Tawa, Shibecha‑cho, Kawakami‑gun, Hokkaido 088‑2339 Japan

Correction to: *Scientific Reports* 10.1038/s41598-021-94505-w, published online 22 July 2021

In the original PDF version of this Article a previous rendition of Figure 1 was published. The original Figure 1 and accompanying legend appear below.

**Figure 1:**
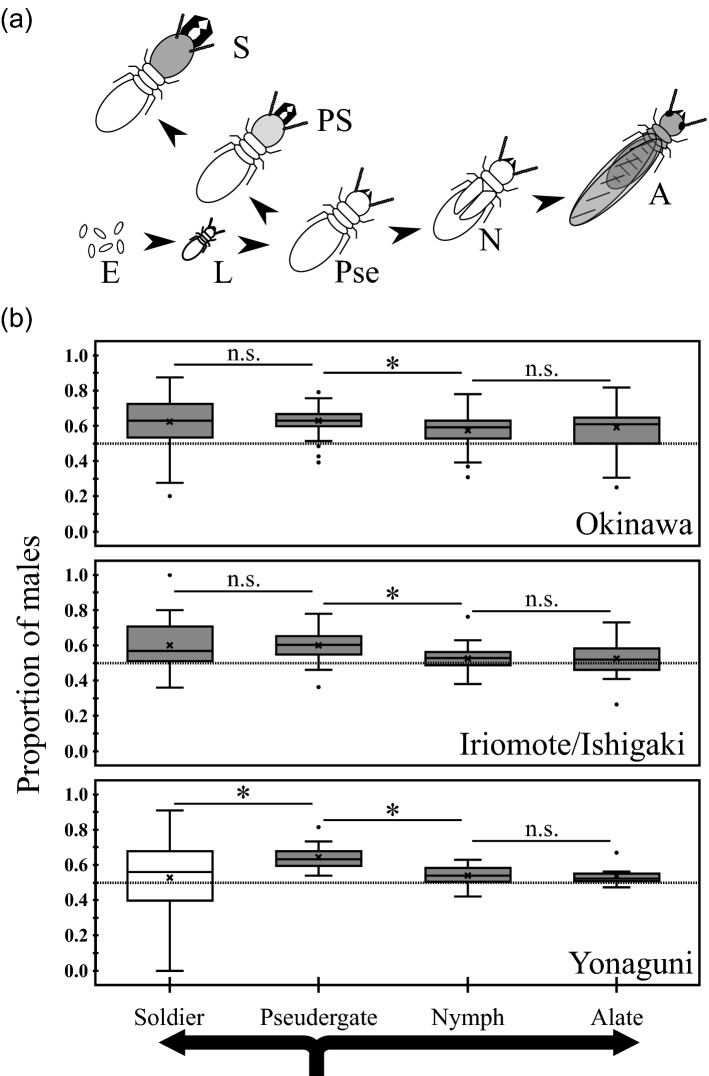
Relationship between caste developmental pathway and numerical sex ratios in the three populations. (**a**) The caste developmental pathway of *Neotermes sugioi*^26^. *E* egg, *L* larva, *Pse* pseudergates, *PS* pre-soldier, *S* soldier, *N* nymph, *A* alate. (**b**) Comparisons of numerical sex ratios between castes. In each box, the cross symbol indicates the mean, bar in the box indicates the median, and the box top and bottom indicate the first and third quartiles, respectively. The asterisk next to each bar between boxes indicates *p* < 0.05 (glmm analysis with sequential Bonferroni), and "n.s." indicates “not significant”, i.e., *p* ≥ 0.05. The gray boxes indicate deviations from equal sex ratios at *p* < 0.05 (Wald tests in glm analysis with sequential Bonferroni), and the white boxes indicate *p* ≥ 0.05.

This error has now been corrected in the PDF version of the Article; the HTML version was correct from the time of publication.

