# Supplementary material for: Caste development and sex ratio of the Ryukyu drywood termite Neotermes sugioi and its potential mechanisms
Source: Sci Rep. 2021 Jul 22;11:15037. doi: 10.1038/s41598-021-94505-w (PMC8298410; doi:10.1038/s41598-021-94505-w)
Supplement: Supplementary file 1 — Supplementary Information. [file 41598_2021_94505_MOESM1_ESM.pdf]

Caste development and sex ratio of the Ryukyu drywood termite *Neotermes sugioi* and its potential mechanisms

Y. Miyaguni [1], A. Agarie [2], K. Sugio [3], K. Tsuji [4], K. Kobayashi [5,] *

[1] Global Education Institute, University of the Ryukyus, Okinawa 903-0213, Japan.

[2] Department of Environmental Science and Conservation Biology, United Graduate School of Agricultural Sciences, Kagoshima University, Kagoshima 890-8580, Japan.

[3] Graduate School of Education, University of the Ryukyus, Nishihara, Okinawa 903-0213, Japan.

[4] Entomological Laboratory, Faculty of Agriculture, University of the Ryukyus, Okinawa 903-0213, Japan.

[5] Hokkaido Forest Research Station, Field Science Education and Research Center, Kyoto University, 553 Tawa, Shibecha-cho, Kawakami-gun, Hokkaido 088-2339, Japan.

* Correspondence: kobayashi.kazuya.3w@kyoto-u.ac.jp

Supplementary Material

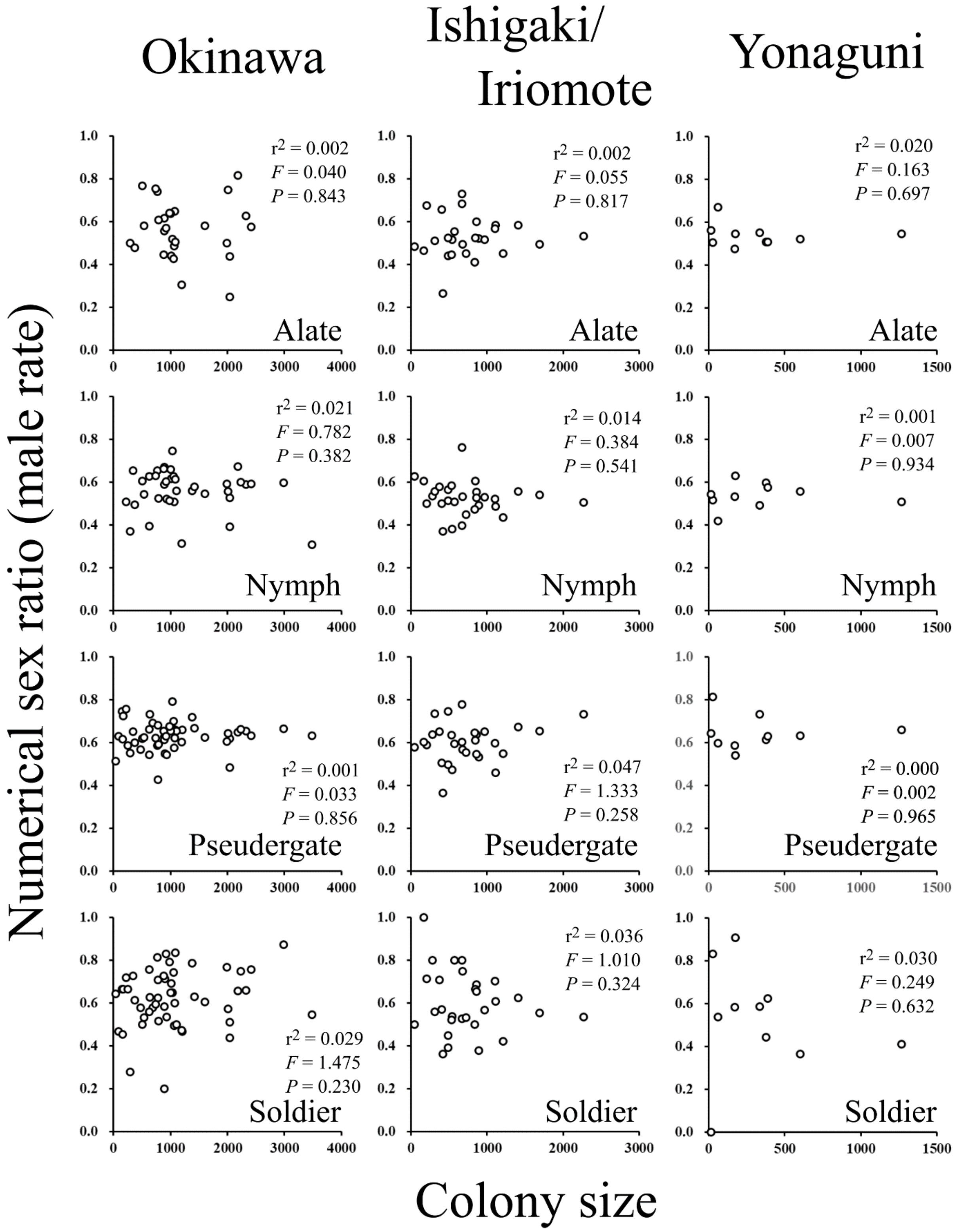

Supplementary Figure 1. Scatter plots of the numerical sex ratio (male rate) and colony size in pseudergates, nymphs, alates, and soldiers of *Neotermes sugioi*. There was no significant correlation between the sex ratio and colony size in any of the castes of any of the populations.

Supplementary Table 1. Detailed composition of the sex ratio of *Neotermes sugioi* colonies in the Okinawa, Ishigaki/Iriomote, and Yonaguni populations (M, male; F, female).

No	Colony	Day	GPS		Popoulation	Reproductive				Colony size (Number of pseudargate)	Caste							
						Adult		Neotenic			Pseudergate		Nymph		Alate		Soldier	
			N	E		F	M	F	M		F	M	F	M	F	M	F	M
1	C104	2010/04/12	26.24850	127.75539	Okinawa	1	1	0	0	3481	110	190*	18	8			15	18
2	C105	2010/05/25	26.23136	127.74531	Okinawa	1	1	0	0	-	182*	118					25	25
3	C106	2010/05/31	26.24842	127.75494	Okinawa	1	1	0	0	-	92	208*	50	150*	10	33*	39	61*
4	C107	2010/06/05	26.16628	127.74831	Okinawa	1	1	0	0	2038	114	186*	133*	86	3	1	45	47
5	C108	2010/06/08	26.15172	127.74858	Okinawa	1	0	0	0	-	116	184*	66	134*	33	135*	23	33
6	C110	2010/06/14	26.25139	127.72919	Okinawa	1	1	0	0	-	138	162	164	236*	80	126*	63	113*
7	C111	2010/08/04	26.21778	127.73136	Okinawa	1	0	0	0	2187	105	195*	94	194*	28	125*	37	71*
8	C112	2010/08/10	26.15567	127.71736	Okinawa	1	1	0	0	-	131	169*	115	157*	15	55*	15	41*
9	C113	2010/08/12	26.24849	127.75475	Okinawa	0	0	0	1	768	124	176*	95	180*	24	69*	5	22*
10	C114	2010/08/14	26.24849	127.75475	Okinawa	1	1	0	0	1070	113	187*	93	149*	14	26	8	12
11	C115	2010/08/16	26.24815	127.75497	Okinawa	0	1	0	0	2043	155	145	97	108	94	73	54	42
12	C116	2010/08/20	26.21794	127.73165	Okinawa	1	1	0	0	2328	104	196*	109	157*	49	83*	78	152*
13	C117	2010/08/23	26.24851	127.75465	Okinawa	1	1	0	0	-	116	184*	-	-	202	286*	12	27*
14	C118	2010/08/25	26.24721	127.78013	Okinawa	1	1	0	0	-	112	188*	-	-	48	85*	12	35*
15	C119	2010/08/29	26.22916	127.76762	Okinawa	1	0	0	1	2230	101	199*	24	36			6	18*
16	C120	2010/09/01	26.21790	127.73135	Okinawa	1	0	0	0	-	113	187*	98	112	114	191*	51	134*
17	C121	2010/09/05	26.22914	127.76726	Okinawa	1	1	0	0	-	94	196*	94	122	60	97*	35	46
18	C126	2011/01/23	26.40063	127.80817	Okinawa	1	1	0	0	147	35	103*					5	10
19	C127	2011/01/23	26.38775	127.84053	Okinawa	1	0	0	0	170	47	123*					5	10
20	C128	2011/01/24	26.38986	127.84086	Okinawa	1	1	0	0	907	180	220					6	15
21	C129	2011/02/01	26.38775	127.84053	Okinawa	1	1	0	0	2007	107	193*	177	223*	3	9	38	51
22	C130	2011/02/06	26.31125	127.91633	Okinawa	1	0	0	0	37	18	19					5	9
23	C132	2011/02/07	26.30075	127.91847	Okinawa	1	1	0	0	687	92	208*					8	11
24	C133	2011/02/08	26.30064	127.91842	Okinawa	1	1	0	0	641	80	220*					22	37
25	C134	2011/02/12	26.30064	127.91842	Okinawa	1	0	0	0	221	52	162*	129	134			14	36*
26	C135	2011/02/21	26.30075	127.91847	Okinawa	1	1	0	0	1210	102	198*					17	15
27	C136	2011/02/25	26.30075	127.91847	Okinawa	1	1	0	0	2987	100	200*	152	226*			2	14*
28	C137	2011/05/27	26.38144	127.81814	Okinawa	1	1	0	0	292	128	158	135*	79	2	2	13	5
29	C138	2011/05/27	26.38142	127.81808	Okinawa	1	0	0	0	778	172	128*					7	17
30	C140	2011/05/28	26.38169	127.81822	Okinawa	1	1	0	0	1380	84	216*	181	229*			21	78*
31	C141	2011/05/29	26.38169	127.81822	Okinawa	1	1	0	0	1069	127	173*	197	203	64	61	51	50
32	C142	2011/05/30	26.38169	127.81822	Okinawa	1	1	0	0	625	137	163	118*	77			18	23
33	C145	2011/05/31	26.25139	127.72919	Okinawa	1	1	0	0	92	34	58*					8	7
34	C147	2011/06/15	26.25894	127.78442	Okinawa	1	1	0	0	255	105	150*					1	2
35	C148	2011/06/15	26.25875	127.78422	Okinawa	1	1	0	0	-	88	212*	44	156*	78	132*	8	9
36	C149	2011/06/15	26.25878	127.78433	Okinawa	1	1	0	0	472	130	170*					8	11
37	C153	2011/06/19	26.25875	127.78428	Okinawa	1	0	0	1	929	137	163	42	46			13	15
38	C155	2011/06/19	26.24831	127.75489	Okinawa	1	1	0	0	164	63	101*					6	5
39	C156	2011/06/20	26.24867	127.75542	Okinawa	1	0	0	1	894	112	188*	135	195*	40	64*	12	3*
40	C171[a]	2012/05/09	26.25808	127.78467	Okinawa	1	1	0	0	1034	215	819*	489	1447*	90	97	20	37*
41	C172[a]	2012/05/10	26.25808	127.78467	Okinawa	0	0	0	0	896	346	550*	640	1308*	51	64	20	28
42	C173	2012/05/16	26.24736	127.75531	Okinawa	1	0	0	1	1985	780	1205*	927	1352*	200	200	30	99*
43	C174	2012/05/18	26.25808	127.78467	Okinawa	1	0	0	0	787	321	466*	409	453	163	254*	29	31
44	C175	2012/05/20	26.24708	127.75625	Okinawa	1	1	0	0	2412	886	1526*	409	596*	145	196*	8	25*
45	C176	2012/05/24	26.24842	127.75494	Okinawa	1	1	0	0	1013	329	684*	259	417*	19	15	16	36*
46	C177	2012/05/28	26.24842	127.75494	Okinawa	1	1	0	0	1053	314	739*	325	549*	4	3	12	35*
47	C178	2012/05/31	26.24842	127.75494	Okinawa	1	0	0	1	510	194	316*	211	326*	3	10	12	12
48	C179	2012/06/01	26.24842	127.75494	Okinawa	1	1	0	0	1090	372	718*	281	445*	93	95	8	41*
49	C180	2012/06/04	26.24789	127.75594	Okinawa	1	1	0	0	776	248	528*					15	25
50	C181	2012/06/06	26.24756	127.75564	Okinawa	1	1	0	0	1424	474	950*	461	630*			20	34

51	C182	2012/06/07	26.24756	127.75558	Okinawa	1	1	0	0	877	304	573*	102	200*	47	38	12	32*
52	C183	2012/06/12	26.24758	127.75567	Okinawa	1	1	0	0	1108	382	726*	200	255*			10	10
53	C184	2012/06/13	26.24814	127.75456	Okinawa	1	1	0	0	626	212	414*	341	573*			9	28*
54	C185	2012/06/14	26.24814	127.75456	Okinawa	1	1	0	0	1001	346	655*	209	408*	21	37*	7	13
55	C186	2012/06/20	26.24814	127.75456	Okinawa	1	1	0	0	927	342	585*	311	475*	167	221*	8	39*
56	C187	2012/06/24	26.25850	127.78444	Okinawa	1	1	0	0	1198	477	721*	216*	99	61*	27	10	9
57	C188	2012/06/24	26.25853	127.78444	Okinawa	1	1	0	0	987	320	667*	16	17	32	57*	6	23*
58	C190	2012/06/27	26.25808	127.78467	Okinawa	1	0	0	1	348	121	227*	46	87*			3	8
59	C193	2012/07/16	26.25808	127.78467	Okinawa	1	1	0	0	378	151	227*	179	175	87	80	12	19
60	C194	2012/07/18	26.25808	127.78467	Okinawa	1	0	0	1	534	200	334*	117	139	90	125*	7	8
61	C195	2012/07/24	26.25808	127.78467	Okinawa	1	1	0	0	1602	600	1002*	412	494*	177	247*	28	43
62	C196	2012/07/19	26.24814	127.75456	Okinawa	1	1	0	0	737	278	459*	271	463*	16	49*	13	19
63	Iri_1	2014/06/26	24.27312	123.83091	Ishigaki/Iriomote	1	1	0	0	1688	585	1103*	955	1128*	285	279	49	61
64	Iri_4	2014/06/26	24.27325	123.83252	Ishigaki/Iriomote	1	0	0	0	833	295	538*	720*	644	387*	269	45	45
65	Iri_6	2014/06/27	24.31971	123.91072	Ishigaki/Iriomote	1	1	0	0	484	123	361*	94	122	49	54	11	9
66	Iri_7	2014/06/27	24.31971	123.91072	Ishigaki/Iriomote	1	0	0	0	968	338	630*	443	498	278	298	32	42
67	Iri_8	2014/06/27	24.31919	123.91009	Ishigaki/Iriomote	0	0	0	0	284	103	181*	252	291			3	12*
68	Ishi_1	2014/06/28	24.38054	124.17516	Ishigaki/Iriomote	1	0	0	0	671	148	523*	66	213*	37	100*	5	20*
69	Ishi_3	2014/06/28	24.37913	124.17416	Ishigaki/Iriomote	1	1	0	0	399	197	202	251	251	53	102*	12	16
70	Ishi_4	2014/06/28	24.45474	124.19125	Ishigaki/Iriomote	1	1	0	0	313	83	230*	208	263*	74	77	11	14
71	Ishi_5	2014/06/28	24.45471	124.19125	Ishigaki/Iriomote	1	1	0	0	1412	461	951*	389	491*	125	175*	32	53*
72	Ishi_6	2014/06/28	24.51544	124.25723	Ishigaki/Iriomote	1	0	0	0	373	130	243*	100	138*			7	17
73	Ishi_8	2014/06/28	24.51354	124.26518	Ishigaki/Iriomote	1	1	0	0	534	194	340*	80	112*	63	51	11	12
74	MY_170529_C2	2017/05/29	24.45485	124.19071	Ishigaki/Iriomote	-	-	-	-	1101	442	659*	398	435	194	254*	16	38*
75	MY_170529_C3	2017/05/29	24.45485	124.19071	Ishigaki/Iriomote	-	-	-	-	207	85	122*	84	84	30	63*	2	5
76	MY_170529_C4	2017/05/29	24.42433	124.23004	Ishigaki/Iriomote	-	-	-	-	572	231	341*	210	218	96	119	3	12*
77	MY_170529_C5	2017/05/29	24.48911	124.27781	Ishigaki/Iriomote	-	-	-	-	166	66	100*	196	300*	157	136	0	5
78	AG_170608_C1	2017/06/08	24.51611	124.25732	Ishigaki/Iriomote	0	0	0	0	536	283	253	390*	241	312	334	6	7
79	AG_170608_C2	2017/06/08	24.48911	124.27781	Ishigaki/Iriomote	1	0	0	0	420	267*	153	183*	108	50*	18	23	13
80	AG_170609_C1	2017/06/09	24.45485	124.49071	Ishigaki/Iriomote	1	1	0	0	887	414	473	340	328	140	153	46*	28
81	AG_170610_C1	2017/06/10	24.50977	124.25738	Ishigaki/Iriomote	1	0	0	0	45	19	26	45	76*	45	42	5	5
82	AG_170610_C2	2017/06/10	24.50977	124.25738	Ishigaki/Iriomote	1	1	0	0	668	266	402*	101*	67	17	37*	9	10
83	AG_170610_C3	2017/06/10	24.50977	124.25738	Ishigaki/Iriomote	1	1	0	0	857	310	547*	50	62	0	0	10	22
84	AG_170815_C4	2017/08/15	24.51611	124.25732	Ishigaki/Iriomote	-	-	-	-	844	328	516*	234	360*	10	11	14	28*
85	AG_180523_2	2018/05/23	24.42566	124.22916	Ishigaki/Iriomote	1	1	0	0	1109	598*	511	478	453	67	94*	18	28
86	AG_180523_6	2018/05/23	24.49910	124.24554	Ishigaki/Iriomote	1	1	0	0	856	388	468*	394	441	2	3	9	17
87	AG_180524_6	2018/05/24	24.49910	124.24554	Ishigaki/Iriomote	1	1	0	0	489	246	243	211	223	14	11	17	11
88	AG_180524_8	2018/05/24	24.49910	124.24554	Ishigaki/Iriomote	0	1	0	0	2271	606	1665*	755	774	227	258	19	22
89	MY_180715_1	2018/07/15	24.45410	124.18827	Ishigaki/Iriomote	1	0	0	0	673	291	382*	133	151	59	58	12	36*
90	MY_180715_4	2018/07/15	24.46462	124.22666	Ishigaki/Iriomote	0	0	0	0	719	321	398*	247*	202	108	89	22	25
91	AG_180717_2	2018/07/17	24.43591	124.21448	Ishigaki/Iriomote	1	1	0	0	1212	547	665*	136	105	72	59	26	19
92	Yo_1	2014/06/24	24.44594	123.01651	Yonaguni	0	0	0	0	376	145	231*	279	417*	194	200	54	43
93	Yo_4	2014/06/24	24.44395	123.00803	Yonaguni	1	0	0	0	169	70	99	62	71	133	120	10	14
94	Yo_5	2014/06/24	24.44514	123.00293	Yonaguni	1	0	0	0	62	25	37	88	64	41	83*	12	14
95	Yo_6	2014/06/24	24.44512	123.00289	Yonaguni	0	0	0	0	1268	432	836*	605	627	98	117	43	30
96	Yo_9	2014/06/24	24.45211	122.98117	Yonaguni	1	1	0	0	389	144	245*	174	238*	75	77	6	10
97	Yo_10	2014/06/24	24.45211	122.98114	Yonaguni	0	0	0	0	27	5	22*	90	96	72	73	1	5
98	Yo_11	2014/06/24	24.44553	122.97396	Yonaguni	1	1	0	0	333	89	244*	265	258	84	103	17	24
99	Yo_13	2014/06/24	24.44554	122.97376	Yonaguni	1	1	0	0	174	80	94	178	303*	51	61	1	10*
100	Yo_14	2014/06/24	24.44553	122.97388	Yonaguni	0	0	0	0	600	221	379*	250	317*	187	203	21	12
101	Yo_15	2014/06/25	24.45637	122.97619	Yonaguni	0	0	0	0	14	5	9	42	50	21	27	3	0
102	Yo_16	2014/06/25	24.45641	122.97616	Yonaguni	1	1	0	0	-	-	-	-	-	363	396	-	-

Colonies with character "a" indicate that colonies showed poor food availability. * indicates that the sex ratio differed significantly from the male:female ratio of 1:1, as determined by a simple binominal test ($p < 0.05$); - indicates accidental loss of data or data that was not recorded; blank cells indicate that the colony did not hold castes.

Supplementary Table 2. Comparison of head width and dry weight between male and female alates of *Neotermes sugioi* in the Okinawa, Ishigaki/Iriomote, and Yonaguni populations.

Colony	Population	Number of individuals measured (F : M)	Head width (mean ± SD, mm)			Dry weight (mean ± SD, 0.0001g)		
			Female	Male	M/F of mean	Female	Male	M/F of mean
c171	Okinawa	20 (10 : 10)	1.800 ± 0.047	1.822 ± 0.042	1.012	55.7 ± 6.0	57.2 ± 2.7	1.027
c172	Okinawa	20 (10 : 10)	1.851 ± 0.052	1.872 ± 0.017	1.012	72.6 ± 6.9	71.6 ± 2.4	0.986
c173	Okinawa	20 (10 : 10)	1.865 ± 0.023	1.874 ± 0.033	1.005	64.9 ± 4.0	63.2 ± 6.1	0.974
c174	Okinawa	20 (10 : 10)	1.809 ± 0.036	1.813 ± 0.043	1.002	59.3 ± 9.5	59.9 ± 8.8	1.010
c175	Okinawa	20 (10 : 10)	1.809 ± 0.015	1.804 ± 0.026	0.997	66.7 ± 4.1	65.3 ± 4.0	0.979
c176	Okinawa	20 (10 : 10)	1.796 ± 0.031	1.798 ± 0.017	1.001	68.3 ± 1.6	66.9 ± 3.1	0.980
c177	Okinawa	7 (4 : 3)	1.819 ± 0.028	1.836 ± 0.016	1.009	76.8 ± 4.0	76.3 ± 1.5	0.995
c178	Okinawa	13 (3 : 10)	1.762 ± 0.013	1.810 ± 0.021	1.027	74.0 ± 5.6	77.3 ± 1.6	1.045
c179	Okinawa	20 (10 : 10)	1.818 ± 0.031	1.844 ± 0.022	1.014	71.2 ± 3.3	69.1 ± 4.7	0.971
c182	Okinawa	20 (10 : 10)	1.773 ± 0.025	1.814 ± 0.032	1.023	55.8 ± 3.5	57.6 ± 4.4	1.032
c185	Okinawa	20 (10 : 10)	1.762 ± 0.017	1.756 ± 0.022	0.996	57.2 ± 3.8	56.2 ± 4.0	0.983
c186	Okinawa	20 (10 : 10)	1.813 ± 0.029	1.818 ± 0.031	1.002	64.6 ± 6.0	67.8 ± 2.6	1.050
c187	Okinawa	20 (10 : 10)	1.800 ± 0.044	1.799 ± 0.048	1.000	63.6 ± 4.5	62.0 ± 3.4	0.975
c188	Okinawa	20 (10 : 10)	1.805 ± 0.036	1.809 ± 0.026	1.002	58.6 ± 5.9	58.1 ± 6.3	0.991
c193	Okinawa	20 (10 : 10)	1.746 ± 0.037	1.775 ± 0.020	1.017	56.7 ± 5.4	54.7 ± 5.5	0.965
c194	Okinawa	19 (9 : 10)	1.804 ± 0.041	1.802 ± 0.025	0.999	56.2 ± 4.4	52.1 ± 5.2	0.927
c195	Okinawa	20 (10 : 10)	1.817 ± 0.036	1.825 ± 0.043	1.004	63.2 ± 4.3	63.6 ± 3.9	1.006
c196	Okinawa	20 (10 : 10)	1.719 ± 0.028	1.752 ± 0.032	1.020	50.0 ± 3.9	52.8 ± 3.9	1.056
				**Mean**	**1.008***		**Mean**	**0.997**
Iri_1	Ishigaki/Iriomote	20 (10 : 10)	1.731 ± 0.034	1.755 ± 0.028	1.013	56.5 ± 8.7	55.0 ± 4.1	0.973
Iri_4	Ishigaki/Iriomote	20 (10 : 10)	1.754 ± 0.027	1.758 ± 0.025	1.002	62.2 ± 4.6	56.8 ± 4.6	0.913
Iri_6	Ishigaki/Iriomote	20 (10 : 10)	1.848 ± 0.042	1.850 ± 0.034	1.001	74.8 ± 8.2	72.4 ± 5.2	0.968
Iri_7	Ishigaki/Iriomote	20 (10 : 10)	1.834 ± 0.042	1.836 ± 0.038	1.001	70.1 ± 6.9	71.9 ± 4.5	1.026
Ishi_1	Ishigaki/Iriomote	20 (10 : 10)	1.708 ± 0.035	1.739 ± 0.044	1.018	49.1 ± 4.7	51.9 ± 6.0	1.057
Ishi_3	Ishigaki/Iriomote	20 (10 : 10)	1.791 ± 0.057	1.808 ± 0.055	1.009	57.3 ± 7.6	56.7 ± 5.1	0.990
Ishi_4	Ishigaki/Iriomote	20 (10 : 10)	1.762 ± 0.046	1.794 ± 0.029	1.018	52.8 ± 4.0	54.1 ± 2.9	1.025
Ishi_5	Ishigaki/Iriomote	20 (10 : 10)	1.813 ± 0.022	1.817 ± 0.041	1.002	60.7 ± 3.0	57.9 ± 7.2	0.954
Ishi_8	Ishigaki/Iriomote	20 (10 : 10)	1.762 ± 0.038	1.755 ± 0.030	0.996	52.4 ± 3.6	51.2 ± 2.0	0.977
				**Mean**	**1.007***		**Mean**	**0.987**
Yo_01	Yonaguni	20 (10 : 10)	1.697 ± 0.037	1.691 ± 0.039	0.997	64.2 ± 6.0	62.6 ± 6.3	0.975
Yo_04	Yonaguni	20 (10 : 10)	1.715 ± 0.040	1.726 ± 0.025	1.007	57.5 ± 5.8	58.6 ± 5.7	1.019
Yo_05	Yonaguni	20 (10 : 10)	1.621 ± 0.041	1.632 ± 0.049	1.007	51.7 ± 3.7	54.4 ± 6.1	1.052
Yo_06	Yonaguni	20 (10 : 10)	1.676 ± 0.027	1.648 ± 0.036	0.983	56.7 ± 4.3	50.7 ± 3.1	0.894
Yo_09	Yonaguni	20 (10 : 10)	1.685 ± 0.029	1.667 ± 0.020	0.989	59.7 ± 4.2	53.9 ± 2.3	0.903
Yo_10	Yonaguni	20 (10 : 10)	1.630 ± 0.042	1.676 ± 0.028	1.028	59.4 ± 6.1	60.0 ± 5.0	1.010
Yo_11	Yonaguni	20 (10 : 10)	1.649 ± 0.040	1.644 ± 0.036	0.997	56.7 ± 2.9	53.0 ± 5.8	0.935
Yo_13	Yonaguni	20 (10 : 10)	1.700 ± 0.024	1.722 ± 0.039	1.013	55.3 ± 6.2	53.3 ± 4.9	0.964
Yo_14	Yonaguni	20 (10 : 10)	1.675 ± 0.040	1.682 ± 0.038	1.005	53.1 ± 6.5	49.8 ± 4.2	0.938
Yo_15	Yonaguni	20 (10 : 10)	1.670 ± 0.018	1.696 ± 0.040	1.015	68.8 ± 4.3	67.3 ± 4.4	0.978
Yo_16	Yonaguni	20 (10 : 10)	1.696 ± 0.044	1.705 ± 0.039	1.005	60.2 ± 4.7	58.4 ± 5.1	0.970
				**Mean**	**1.004**		**Mean**	**0.967***

* indicates a statistically significant difference in body size between males and females.

Supplementary Table 3. Detailed results of the regression analysis of the total biomass of alates on that of male or female alates in the colonies of *Neotermes sugioi*.

Dependent variables		Estimate	S.E.	*t*-value	*p*-value
Female of Okinawa					
	Intercept	22.59	29.04	0.78	0.448
	Coefficient	0.43	0.02	21.61	<.0001
Male of Okinawa					
	Intercept	-22.57	29.04	-0.78	0.4483
	Coefficient	0.57	0.02	28.77	<.0001
Female of Ishigaki/Iriomote					
	Intercept	-120.13	94.83	-1.27	0.2457
	Coefficient	0.56	0.04	13.71	<.0001
Male of Ishigaki/Iriomote					
	Intercept	120.15	94.85	1.27	0.2458
	Coefficient	0.44	0.04	10.67	<.0001
Female of Yonaguni					
	Intercept	-16.95	20.81	-0.81	0.4365
	Coefficient	0.50	0.01	43.96	<.0001
Male of Yonaguni					
	Intercept	16.93	20.82	0.81	0.4369
	Coefficient	0.50	0.01	44.64	<.0001

S.E.: Standard Error

Supplementary Table 4. Neotenic system in *Neotermes sugioi* collected from the Ishigaki/Iriomote and Yonaguni populations.

Colony	Population	Island	Number of subcolonies reared	Number of subcolonies that produced neotenics	
				Male neotenic	Female neotenic
Iri_2	Ishigaki/Iriomote	Iriomote	3	3	0
Iri_3	Ishigaki/Iriomote	Iriomote	3	2	0
Ishi_2	Ishigaki/Iriomote	Ishigaki	3	2	0
Ishi_7	Ishigaki/Iriomote	Ishigaki	3	2	0
Yo_2	Yonaguni	Yonaguni	3	2	0
Yo_7	Yonaguni	Yonaguni	3	2	0
Yo_8	Yonaguni	Yonaguni	3	2	0

Experimental colonies of *N. sugioi* sampled from Ishigaki/Iriomote and Yonaguni populations produced only male neotenics. We collected seven colonies of *N. sugioi* on the three islands in June 2014: three from Yonaguni Island (Yonaguni population), two from Ishigaki, and two from Iriomote Island (Ishigaki/Iriomote population). The colonies were cut into small blocks, and all colony members, including reproductives, were collected. The collected individuals were classified into the following castes [45]: small larvae, pseudergates, nymphs, pre-alate nymphs, alates, pre-soldiers, soldiers, the queen (up to one individual in each colony), and the king (up to one individual in each colony). From each colony, we established three sub-colonies, each of which consisted of no reproductives and 50 pseudergates that were randomly sampled but always included both males and females. These sub-colonies were maintained for 40 days according to the methods described in a previous study [16].
